# Prolonged Survival With Homozygous Deletion of Exon 9 in Perlman Syndrome: A Case Report

**DOI:** 10.1155/crig/9916711

**Published:** 2026-04-25

**Authors:** Esther Levy, Leighton Elliott, Michal A. Miller, Mackenzie Kramer

**Affiliations:** ^1^ Hematology/Oncology Fellowship Program, Geisinger Medical Center, Danville, Pennsylvania, USA, geisinger.edu; ^2^ Division of Hematology and Oncology, University of Florida, Gainesville, Florida, USA, ufl.edu; ^3^ Pediatric Hematology and Oncology, Janet Weis Children’s Hospital, Danville, Pennsylvania, USA; ^4^ Internal Medicine, Abington Jefferson Health, Abington, Pennsylvania, USA, abingtonhealth.org

**Keywords:** DIS3L2, nephroblastomatosis, Perlman, renal dysplasia, Wilms

## Abstract

Perlman syndrome is a rare autosomal recessive overgrowth disorder characterized by macrosomia, nephromegaly, renal dysplasia, and characteristic facial features. It has both similarities and differences to other more common overgrowth syndromes. Pathogenic homozygosity is extremely rare in nonconsanguineous relationships. Survival is predicted by differences among various germline mutations in the DIS3L2 gene on chromosome 2q37.1, with prolonged survival documented in heterozygous mutations allowing partial exoribonuclease function. Although homozygous deletions of exon 9 are rare and have been associated with poor survival, we describe a case report of the only known patient with Perlman syndrome to live past 2 years old with this deletion. Diligent management and surveillance may be associated with prolonged survival in Perlman syndrome.

## 1. Introduction

Perlman syndrome was initially described by Liban and Kozenitzky in two siblings of a consanguineous Yemenite Jewish family with further characterization by Perlman et al. [1]. Most recently, molecular genetics and long‐term survival have been described with various germline mutations in the DIS3L2 gene on chromosome 2q37.1, with homozygous mutations conferring decreased survival. While most infants die within the first year of life, heterozygous mutations allowing partial exonuclease function may contribute to prolonged survival, with a case report discussing such in a 6‐year‐old Japanese female [2]. However, homozygous deletions of exon 9 have been associated with poor survival, with the oldest documented patient living to 9 months old [3, 4].

## 2. Case Description

We present the case of a 32‐month‐old male born at 36 weeks gestation. He required admission to the neonatal intensive care unit for respiratory support and was found to have fetal gigantism, pulmonary hypoplasia, enlarged kidneys, and focal epilepsy.

The patient’s sister died at 3 hours of life. She was born via elective cesarean section at 36 weeks and 1 day gestation due to multiple congenital anomalies found prenatally via ultrasound including anhydramnios from at least 27 weeks, VSD with biventricular hypertrophy and pericardial effusion, and bilateral polycystic kidney disease. Due to prolonged anhydramnios, she was delivered in respiratory failure requiring intubation and oscillating ventilation and was given surfactant with no appreciable response. Her parents made the decision to provide ventilation only without chest compressions or cardiac medications. The patient was born at 3541 grams (g), 46 centimeters (cm) long, and had a 33.6 cm head circumference. Her weight for gestational age was in the World Health Organization (WHO) 88.3^rd^ percentile, and her head circumference was greater than the WHO 97.5^th^ percentile [5]. Physical exam described an extremely malformed large‐for‐gestational‐age infant with no spontaneous movements other than occasional grasping, a flat facies with swelling, a distended abdomen, malformed and edematous lower extremities, mottled skin with extensive bruising, and muscular laxity. Her APGAR scores were 1, 2, and 2 at 1, 5, and 10 minutes, respectively. She was suspected to have autosomal recessive polycystic kidney disease at birth. However, testing after birth showed a negative PKHD1 sequencing and deletion/duplication analysis with a normal SNP array. Further testing was not performed as she became hypoxic and died shortly after birth.

The patient’s prenatal history included ultrasonographic findings of bilateral enlarged kidneys, a pericardial effusion with an echogenic intracardiac focus, choroid plexus cysts, and oligohydramnios which started at 24 weeks gestation. He was born via cesarean section at 36 weeks and 6 days gestation without respiratory effort requiring positive pressure ventilation followed by continuous positive airway pressure. The patient was born weighing 3690 g, was 49 cm long, and had a 37 cm head circumference. His weight for gestational age was in the WHO 96.9^th^ percentile and his head circumference was greater than the WHO 97.5^th^ percentile [5]. Physical exam described an infant with facial swelling, a neck with redundant nuchal folds, the appearance of webbing on the right side of the occipital bone, and no further malformations. At that time, the neonatologist did not suspect a specific syndrome due to a lack of other malformations present. However, due to his bilateral enlarged kidneys, and a family history concerning for a possible syndrome, whole exome sequencing was sent on the patient. This sequencing was normal, however, there remained a high suspicion of a genetic syndrome, therefore a chromosomal SNP microarray was sent.

The patient’s chromosomal SNP microarray showed a homozygous deletion of an 18‐kilobase region within chromosome 2q37.1 (arr [hg19 2q37.1 (233,021,151–233,039,557) × 0). Deletions in the gene encoded, the DIS3L2 gene, are associated with autosomal recessive Perlman syndrome.

Perlman syndrome, a type of overgrowth syndrome, has many similarities to other overgrowth syndromes, such as facial dysmorphisms, generalized, segmental, or visceral overgrowth, developmental delays, and tumor risk. Micrognathia was not noted on initial physical examinations of this patient, but became prevalent at 2 months of life and began to obstruct his airway, causing obstructive sleep apnea (OSA) requiring mandibular distraction, with successful improvement in feeding and apnea. He did not have other facial dysmorphisms. He was found to have bilateral cryptorchidism and bilateral renomegaly but he did not have macroglossia, hepatomegaly, or hypoglycemia. He had a severe developmental delay and an increased tumor risk.

The patient′s neurologic course involved focal seizures, developmental delays, plagiocephaly, and hypotonia. He had a brain MRI shortly after birth which showed diffuse prominent extra‐axial spaces, underopercularization of Sylvian fissures, a thin corpus callosum, prominent ventral horns, asymmetry of the cerebral hemispheres, bilateral choroid plexus cysts, and dolichocephaly with biconcave frontal bones. His focal seizures were described as staring with his eyes fixed in one direction, his lips turning blue, and his oxygen level decreasing. He required levetiracetam, oxcarbazepine, and gabapentin to control his focal seizures so that they occurred monthly. He was involved with physical and occupational therapy for his global, significant, severe developmental delays, including cognitive, communicative, adaptive, and motor delays. Shortly before his death at 32 months of age, he was sitting on his own, clapping, tracking others, and following simple commands.

Oncologic clinical course for this patient involved the identification of bilateral nephroblastomatosis at the age of 6 months via ultrasound surveillance. This was treated with chemotherapy with the National Wilms Tumor Study‐5 protocol with vincristine and dactinomycin. However, at 16 months old, he experienced progression of his nephroblastomatosis on the left side requiring surgical intervention. Partial left nephrectomy of the nephroblastoma (Wilms tumor) showed favorable histology with negative margins. He subsequently received adjuvant dose reduced chemotherapy with vincristine, doxorubicin, cyclophosphamide and etoposide. One year later, surveillance imaging showed concern for a mass on the right side, requiring a right radical nephrectomy which showed metachronous Wilms tumor with diffuse anaplasia. He then received adjuvant chemotherapy with vincristine and dactinomycin. Repeat left partial nephrectomy 3 months later revealed diffuse anaplasia with positive margins. The patient then began a planned treatment break from chemotherapy with surveillance imaging.

At 32 months of age, the patient developed respiratory distress with acute hypoxic respiratory failure and sepsis presumed to be due to pyelonephritis and pneumonia. He was found to have a new lung metastasis from his Wilms tumor, at which time his parents decided not to pursue further radiation and were informed that he had no further chemotherapy options. He was discharged on hospice and died 1 week later.

## 3. Discussion

Perlman syndrome is an extremely rare autosomal recessive genetic defect categorized as an overgrowth syndrome that causes death to most patients in infancy.

There are many overgrowth syndromes with distinct features, although they all include dysregulated somatic growth due to genetic changes. The most common overgrowth syndromes, Beckwith–Wiedemann syndrome and Sotos syndrome, may be confused with Perlman syndrome, however they have several distinctions. Beckwith–Wiedemann syndrome is the most common overgrowth syndrome and is characterized by overgrowth, polyhydramnios, abdominal wall defects at birth, macroglossia, neonatal hyperinsulinemic hypoglycemia, visceromegaly, hemihyperplasia, facial dysmorphology, and predisposition for embryonal tumors. Sotos syndrome is characterized by childhood overgrowth with tall stature, macrocephaly, facial dysmorphology, and developmental delay [6]. Alternatively, Perlman syndrome typically presents with polyhydramnios, macrosomia, nephromegaly, Wilms tumor, hepatomegaly, cryptorchidism, and facial dysmorphology [7]. Some key differences in this syndrome are the lack of abdominal wall defects, macroglossia, neonatal hyperinsulinemia hypoglycemia, and hemihyperplasia seen in Beckwith–Wiedemann syndrome and the prenatal overgrowth which contrasts with childhood overgrowth seen in Sotos syndrome. The patient had many of the classical features of Perlman syndrome, although he did not have facial dysmorphology, hepatomegaly, or polyhydramnios typical of the disease.

Various factors contribute to mortality in Perlman syndrome: most notably respiratory and renal failure associated with pulmonary hypoplasia and renal dysplasia. Dysplastic medullary parenchyma develops into nephroblastomatosis and hamartomas which develop into Wilms tumor among nephrogenic rests [1]. Neonatal mortality is high mainly due to renal disease, and most patients who survive the neonatal period develop Wilms tumor due to residual embryonic nephrogenic tissue [2, 8].

**FIGURE 1:**
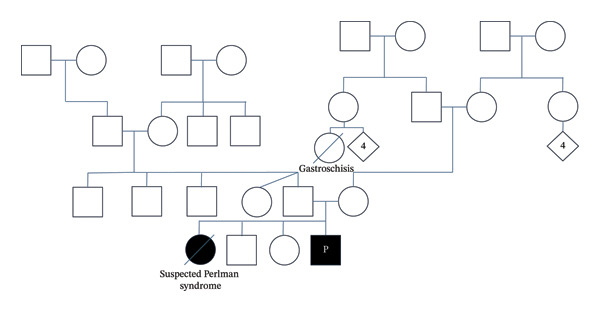
Pedigree of patient’s family.

Pathogenic homozygosity in this patient was a rare happenstance. The patient’s parents had no consanguinity or Ashkenazi Jewish heritage. The mother’s family was Polish and German, and the father’s family was German. Aside from the patient’s deceased sister, there was no family history of similar findings. Both parents had normal renal ultrasounds. The patient had a cousin once removed who died as a baby with gastroschisis, which can be associated with Beckwith–Wiedemann syndrome but is not typical in Perlman syndrome. He also notably had a sister with concern for Perlman syndrome and no known consanguinity, although she was never formally diagnosed (Figure 1). De‐novo mutations are possible but rare and unlikely to be a factor in this patient’s genetic blueprint because his deceased sister also had findings suggestive of Perlman syndrome.

The prevalence of Perlman syndrome worldwide is approximately 1 in 1 million individuals [9]. The carrier frequency in European, non‐Finnish populations, however, is about 1 in 1233 individuals [10]. Given this carrier frequency, the estimated prevalence in this population using the Hardy–Weinberg principle is about 1 in 6 million individuals. Therefore, it was a rare event that both parents had heterozygosity.

Perlman syndrome is characterized by the DIS3L2 gene mutation. This mutation results in the loss of function of exoribonuclease activity, leading to dysregulated tumorigenesis [11]. DIS3L2 is a negative regulator of cell growth as it leads to apoptotic mRNA through the decay of the tumor suppressor, uridylated pre‐let‐7 [12]. Mutations in this gene inhibit apoptotic mRNA decay, translation arrest, and cell death, therefore leading to overgrowth [13]. Homozygous deletions are more severe than heterozygous deletions due to partial exonuclease function in heterozygous patients [2]. This patient’s homozygous deletion of exon 9 on the DIS3L2 gene likely contributed to the chemotherapy‐refractoriness of his Wilms tumor. Regardless, he is the only known patient to live past 9 months old with this mutation, with case reports of patients living until the age of 1 day, 6 months, 8 months, and 9 months [3, 4]. Of note, his sister died at 3 days of age due to suspected Perlman syndrome, suggesting the aggressiveness of his known gene mutation.

The reasons for this patient’s prolonged survival may be related to vigilant clinical surveillance, with aggressive medical therapies including the early resection of Wilms tumors, the recognition of OSA with subsequent mandibular distraction, and a multidisciplinary approach to his healthcare. For patients who are medically fragile, there may be a reluctance to undertake aggressive therapeutic interventions. However, he tolerated chemotherapy and multiple invasive surgeries including sequential partial nephrectomies as well as a mandibular distraction in the treatment of the frequently fatal OSA associated with Perlman’s syndrome.

This patient’s care was managed concurrently between two different academic medical centers, each with their own children’s hospital and various experts. This team‐based approach to his care was instrumental to his prolonged survival since the local children’s hospital, located in and serving a rural population, had limited access to pediatric nephrology consultative services at that time. The local hospital was able to otherwise provide expert pediatric care, decreasing the social burden on the family to travel over 150 miles to the next children’s hospital. This patient’s case serves as a reminder of the importance of ongoing collaborative efforts between multiple hospital systems.

In conclusion, this patient’s case describes both unique and classic features of Perlman syndrome, a rare type of overgrowth syndrome. It also describes the rarity of an inherited mutation from carrier parents without consanguinity in a population where this disease is extremely rare. Additionally, it describes that vigilant clinical surveillance, early aggressive medical therapies, and collaboration between major pediatric centers may aid in the prolongation of survival in this fatal genetic disorder.

## Author Contributions

M.A.M. was responsible for the patient’s clinical management. E.L., L.E., and M.K. collected the data and wrote the initial draft. E.L. contributed to the literature review and all revisions of the manuscript. E.L. edited the final manuscript.

## Funding

No external funding support was obtained.

## Consent

All the patients allowed personal data processing and informed consent was obtained from all individual participants included in the study.

## Conflicts of Interest

The authors declare no conflicts of interest.

## Data Availability

Data sharing is not applicable to this article as no datasets were generated or analyzed during the current study.
